# Protective mAbs and Cross-Reactive mAbs Raised by Immunization with Engineered Marburg Virus GPs

**DOI:** 10.1371/journal.ppat.1005016

**Published:** 2015-06-26

**Authors:** Marnie L. Fusco, Takao Hashiguchi, Robyn Cassan, Julia E. Biggins, Charles D. Murin, Kelly L. Warfield, Sheng Li, Frederick W. Holtsberg, Sergey Shulenin, Hong Vu, Gene G. Olinger, Do H. Kim, Kevin J. Whaley, Larry Zeitlin, Andrew B. Ward, Cory Nykiforuk, M. Javad Aman, Jody Berry, Erica Ollmann Saphire

**Affiliations:** 1 Department of Immunology and Microbial Science, The Scripps Research Institute, La Jolla, California, United States of America; 2 Department of Virology, Faculty of Medicine, Kyushu University, Fukuoka, Japan; 3 Emergent BioSolutions (formerly Cangene Corporation), Winnipeg, Manitoba, Canada; 4 Division of Virology, United States Army Research Institute for Infectious Disease, Ft. Detrick, Maryland, United States of America; 5 Integrated Biotherapeutics, Inc., Gaithersburg, Maryland, United States of America; 6 Department of Integrative Structural and Computational Biology, The Scripps Research Institute, La Jolla, California, United States of America; 7 Department of Medicine, University of California San Diego, La Jolla, California, United States of America; 8 Integrated Research Facility, NIAID, National Institutes of Health, Frederick, Maryland, United States of America; 9 Mapp Biopharmaceutical, Inc., San Diego, California, United States of America; 10 Skaggs Institute for Chemical Biology, The Scripps Research Institute, La Jolla, California, United States of America; Mount Sinai School of Medicine, UNITED STATES

## Abstract

The filoviruses, which include the marburg- and ebolaviruses, have caused multiple outbreaks among humans this decade. Antibodies against the filovirus surface glycoprotein (GP) have been shown to provide life-saving therapy in nonhuman primates, but such antibodies are generally virus-specific. Many monoclonal antibodies (mAbs) have been described against Ebola virus. In contrast, relatively few have been described against Marburg virus. Here we present ten mAbs elicited by immunization of mice using recombinant mucin-deleted GPs from different Marburg virus (MARV) strains. Surprisingly, two of the mAbs raised against MARV GP also cross-react with the mucin-deleted GP cores of all tested ebolaviruses (Ebola, Sudan, Bundibugyo, Reston), but these epitopes are masked differently by the mucin-like domains themselves. The most efficacious mAbs in this panel were found to recognize a novel “wing” feature on the GP2 subunit that is unique to Marburg and does not exist in Ebola. Two of these anti-wing antibodies confer 90 and 100% protection, respectively, one hour post-exposure in mice challenged with MARV.

## Author Summary

The filoviruses have caused multiple outbreaks among humans this decade, including a 90% lethal outbreak of Marburg virus in Angola and a significant, sustained outbreak of Ebola virus in West Africa. The viral surface glycoprotein (GP), which enables filoviruses to infect host cells, is the primary target of the immune system. Antibodies that target filovirus GP have been shown to provide life-saving therapy in nonhuman primates. However, the majority of known antibodies are only reactive against Ebola virus and not other emerging filoviruses. In this study, we present ten antibodies against Marburg virus, elicited by immunization of mice using engineered forms of its GP. Surprisingly, two antibodies exhibit some cross-reactivity to ebolaviruses (including species Ebola, Sudan, Bundibugyo, Reston). Other antibodies in this panel recognize a novel “wing” feature on a portion of GP that is unique to Marburg and does not exist in ebolaviruses, and protect 90%-100% of mice from lethal exposure. These antibodies, and their structural and functional analysis presented here, illuminate directions forward for therapeutics against Marburg virus.

## Introduction

Filoviruses are filamentous, enveloped viruses that can cause highly lethal hemorrhagic fever in both humans and non-human primates. The filovirus family includes the major genera *ebolavirus* and *marburgvirus* and the newly discovered *cuevavirus*. In the *ebolavirus* genus are five known species: Ebola virus (EBOV), Sudan virus (SUDV), Bundibugyo virus (BDBV), Reston virus (RESTV), and Taï Forest virus (TAFV). In the *marburgvirus* genus, there is one species, the eponymously named Marburg virus (MARV) [1]. MARV is further subdivided into different strains, including Ci67, Musoke, Ravn and Angola. Ravn is the most divergent strain of MARV, differing by 21% in genomic sequence from other Marburg strains [2], and is sometimes referenced as a separate filovirus species.

Marburg virus was the first filovirus to be identified when it sickened laboratory workers handling infected animals originating from Uganda in 1967 [3–5]. Marburg virus has since re-emerged at least 8 times, and has been imported to the United States and Europe by travelers who became infected in Africa [6–9]. Angola, the most lethal strain of Marburg virus [10], emerged in 2004 and caused the largest MARV outbreak known to date with an extremely high case fatality rate of 88% [11]. The emergence of Ebola virus in West Africa in 2014 has caused an outbreak unprecedented in magnitude, and is a grim reminder of the devastation that can be caused by filoviruses.

The filoviruses present a single viral protein on their envelope surface, the glycoprotein (GP), which is responsible for attachment and entry of viruses into target cells. GP is expressed as a precursor that is cleaved by furin in the producer cell to yield two subunits: GP1 and GP2, which remain linked by a disulfide bond [12,13]. GP1 contains the putative receptor-binding region [14], as well as two heavily glycosylated domains: a glycan cap which sits immediately atop the putative receptor-binding site and a larger, largely unstructured mucin-like domain [15,16]. The mucin-like domains contain a dense clustering of N- and O-linked glycans and likely mask the GP from immune surveillance [17,18]. The second subunit of GP, termed GP2, possesses the transmembrane domain that anchors GP into the viral surface and the hydrophobic fusion peptide required for fusion. In ebolaviruses, the furin cleavage site lies at residue 501 and the entire mucin-like domain is attached to the GP1 subunit. In Marburg virus, however, the furin cleavage site lies at residue 435, splitting the mucin-like domain so that a portion of it remains attached to the GP2 subunit [19]. We have termed this 66 amino-acid N-terminal GP2 extension the “GP2 wing”.

After cell entry by macropinocytosis [20,21] filovirus GP undergoes additional cleavage by host cathepsin proteases in the endosome [22,23]. This cleavage event removes the glycan cap and the mucin-like domain, resulting in a loss of over 70% of the molecular mass of GP [23–25]. Endosomal cleavage renders GP competent for receptor binding [22,26,27], allowing the exposed GP1 head to bind a shared filovirus receptor, Niemann-Pick C1 (NPC1) [28,29]. Although antibodies that broadly cross-react among ebola- and marburgvirus GPs would be highly desirable, only one such antibody, MR72, has been described [30].

Recent work in non-human primates has demonstrated that passive administration of monoclonal antibody (mAb) cocktails against GP can provide highly effective post-exposure therapy for EBOV infection [31–35]. Polyclonal sera against Marburg virus has shown similar efficacy, suggesting that antibodies could also be a viable treatment option for MARV infection [36]. However, fewer monoclonal antibodies, from which such cocktails could be developed, are currently available for MARV. One human survivor panel has recently been described; most of these mAbs compete for the same site on the GP1 core [16,30]. Antibodies targeting other epitopes on Marburg GP would be desirable in order to form a treatment cocktail.

In general, monoclonal antibody cocktails are most effective when the component antibodies display synergistic effects. Combining mAbs with non-overlapping epitopes can significantly increase the overall potency of the cocktail over the individual mAbs alone [37], and can mitigate antigenic escape by the virus [38,39]. Anti-viral antibodies are often selected based on neutralization, or the ability of the mAbs to prevent viral entry into target cells *in vitro*. However, for filoviruses as well as other viruses, neutralization *in vitro* does not necessarily correlate with protection *in vivo* [40,41]. Non-neutralizing antibodies are known to confer protection by antibody-dependent cellular cytotoxicity (ADCC), phagocytosis, prevention of virus budding, and other mechanisms [42,43]. Indeed, one successful anti-EBOV oligoclonal cocktail is composed entirely of antibodies that are not potent neutralizers [32,44].

In this study we produced a diverse panel of antibodies against Marburg virus by immunization of mice with different strains of the surface GP antigen. Immunogens included GP1-mucin-deleted ectodomains (GPΔmuc) from Marburg strains Ci67, Musoke, Angola, and Ravn. Mucin-deleted immunogens were used to direct the immune response away from the highly variable mucin-like domains. Ten antibodies were chosen and analyzed for *in vitro* neutralization, *in vivo* efficacy, and biochemical recognition of MARV and EBOV GPs. Antibodies against multiple epitopes were found. Four antibodies target a novel MARV-specific “wing” epitope on GP2 (30G3, 30G4, 30G5 and 54G2), and confer 60–100% protection in mice challenged with MARV. A separate MARV-specific antibody, 9A11, directed against an epitope in GP1, confers 65% protection. Another mAb directed against GP1, 40G1, confers 40% protection and was found to be broadly cross-reactive among the core of filovirus GPs, including both marburg- and ebolaviruses.

## Results

### Antibody generation

**Fig 1:**
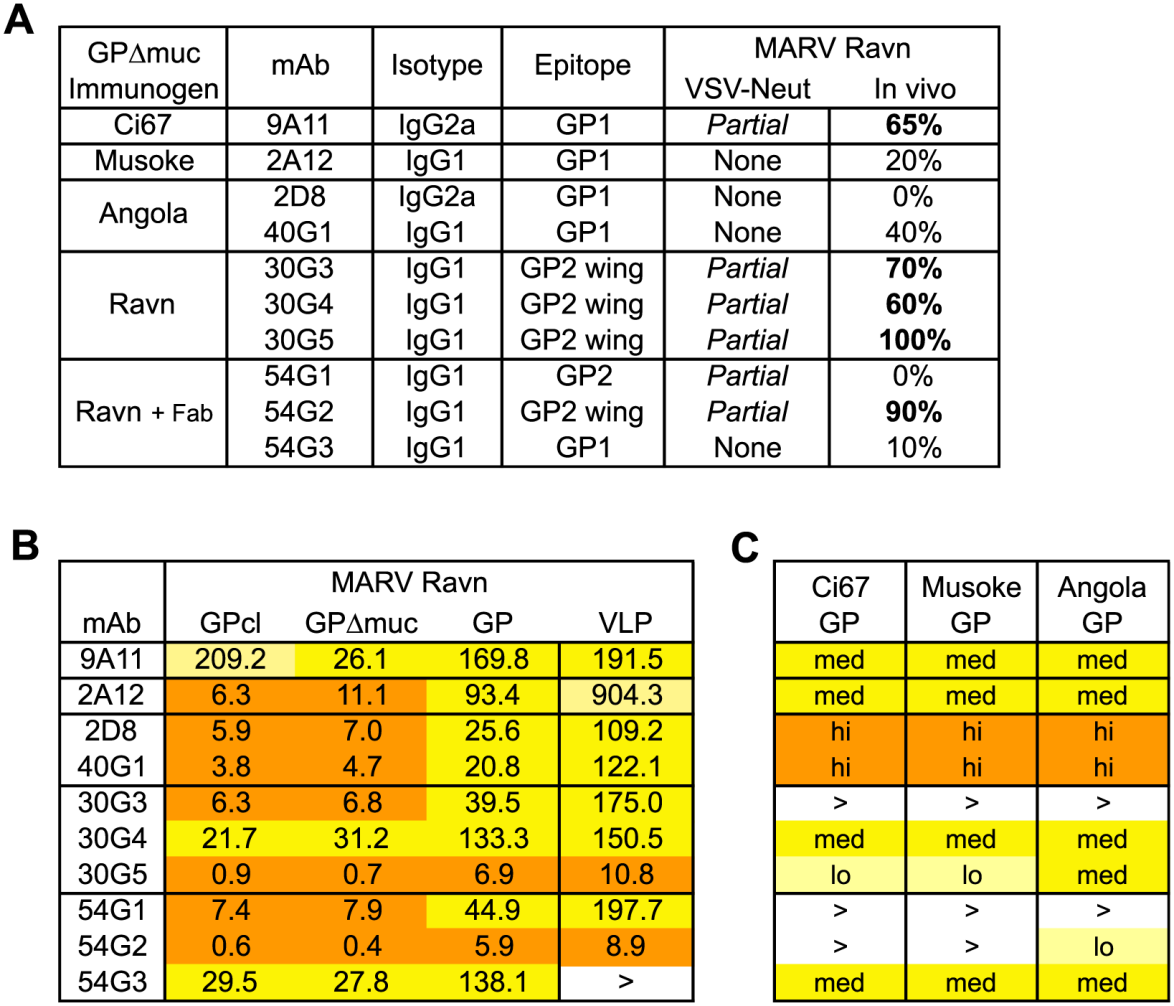
Antibody characterization. (A) Overall summary of mAb generation, characterization, *in vitro* neutralization of MARV GP-pseudotyped VSV, and *in vivo* protection in mice. Antibodies were generated from a single immunogen with the exception of 54G1, 54G2 and 54G3 which were primed with Ravn GPΔmuc and boosted with GPΔmuc 30G4 Fab complex. (B) ELISA EC50 values against different forms of purified MARV Ravn GP or against VLPs in ng/ml. Relative potency is indicated by orange highlight (high, EC50 <20ng/ml), dark yellow (medium, EC50 20-200ng/ml), or light yellow (low, EC50 >200ng/ml). (C) Relative ELISA binding to purified mucin-containing GPs from other MARV strains. No binding at 10μg/ml maximum mAb concentration is represented by >.

To generate MARV GP-specific mAbs, BALB/c mice were immunized with GPΔmuc antigens from either MARV strain Ci67, Musoke, Angola, or Ravn (Fig 1A). Mice for each subset were immunized and boosted with the same antigen with the exception of the 54 series (54G1, 54G2, 54G3). Eight of the ten mAbs in the panel are mouse IgG1. The remaining two mAbs, 9A11 and 2D8, are IgG2a (Fig 1A).

### Antibody binding

**Fig 2:**
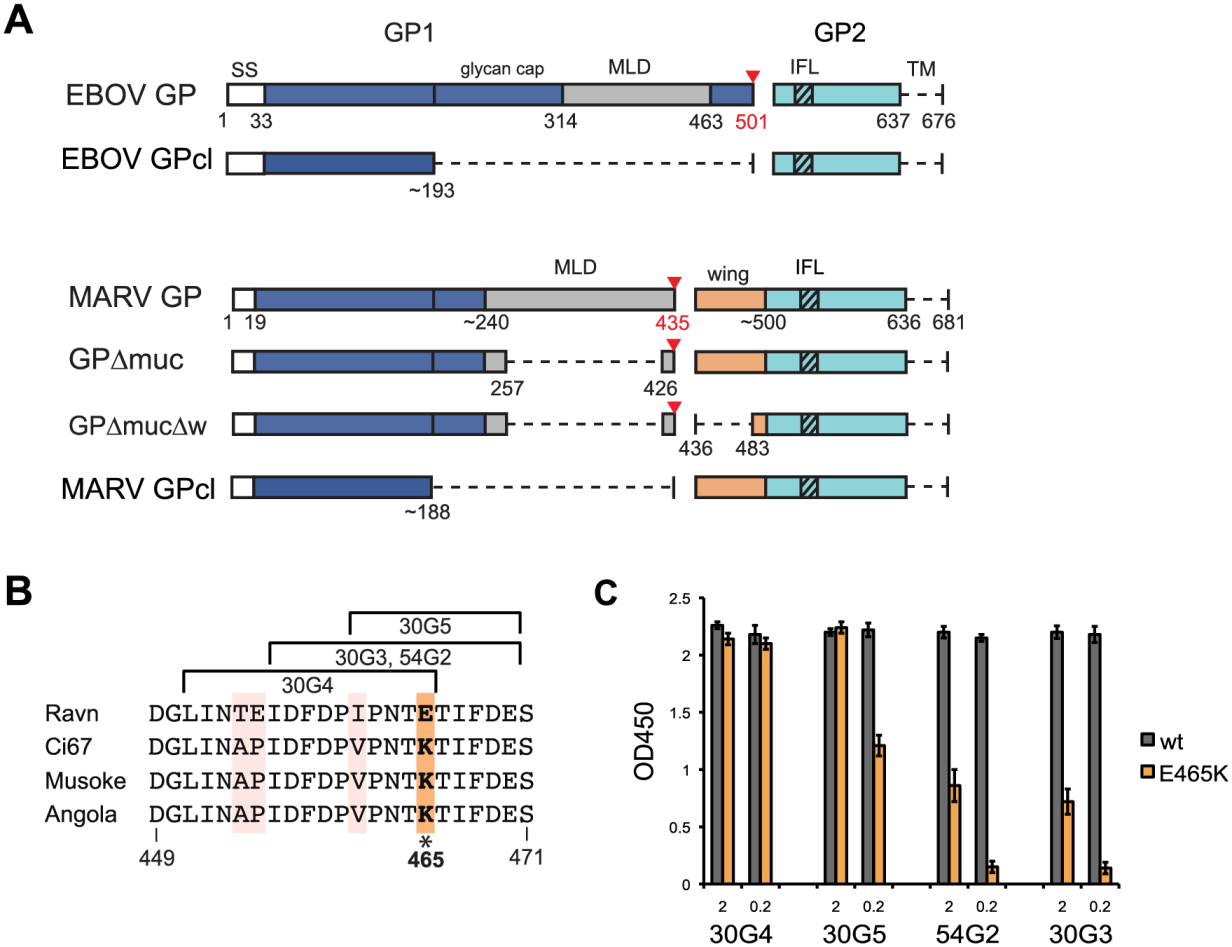
GP schematic and GP2-wing epitope analysis. (A) Schematic of purified GP ectodomains used in this study. Dashed lines represent deleted regions. SS, signal sequence; MLD, mucin-like domain; IFL, internal fusion loop; TM, transmembrane domain. A red triangle indicates the furin cleavage site, numbered in red. The GP2-wing region, which is unique to MARV, is colored orange. B) MARV sequence alignment of pepscan defined epitopes for anti-GP2 wing mAbs. This region has four residues unique to strain Ravn; notably, 465E is 465K in other strains. (C) ELISA binding of GP2-wing mAbs to wild type (wt) and E465K Ravn GPΔmuc at 2 and 0.2μg/ml.

To characterize the binding of mAbs, we performed enzyme-linked immunosorbent assays (ELISAs) with recombinant GPs from four MARV strains, and determined EC50 values for binding with different forms of MARV Ravn GP: GP, GPΔmuc, GPcl (Fig 2A). All ten mAbs exhibit medium binding (EC50 between 20ng/ml and 200ng/ml, colored dark yellow) to high binding (EC50 <20ng/ml, colored orange) against Ravn GP and GPΔmuc (Fig 1B), but only seven of the mAbs cross-react with GP from other MARV strains (Fig 1C). All mAbs bind the protease-cleaved Ravn GP core, termed GPcl, as well as GPΔmuc, with the exception of 9A11. Antibody 9A11 exhibits an 8-fold decrease in binding to GPcl as compared to GPΔmuc (Fig 1B). Additionally, to evaluate whether the mAbs have the capacity to bind cell-surface GP, ELISAs were performed with virus-like particles (VLPs) bearing full-length wild-type MARV Ravn GP. Eight mAbs bind as well (or nearly as well) to VLPs as purified recombinant Ravn GP. In contrast, 2A12 exhibits nearly 10-fold weaker binding to VLPs than to GP ectodomain, and 54G3 binding to VLPs is lost at the highest concentration tested (Fig 1B).

### Epitope determination

To determine antibody epitopes, we performed western blotting with Ravn GP and pepscan analysis with overlapping 15-mer pins of peptides from Ravn or Musoke GP. Five of the mAbs bind GP1, and five bind GP2 by western blot (S1 Fig panel A). Pepscan identified linear epitopes for only four mAbs, 30G3, 30G4, 30G5 and 54G2, all of which overlap within residues 451–471 (Fig 2B). This shared region lies in an extension of GP2 that is specific to MARV (as a result of the furin cleavage site shift from 501 in EBOV to 435 in MARV), which we have termed the GP2 wing (Fig 2A). In order to confirm the pepscan results, we engineered a GPΔmuc with an additional deletion of residues 436–483, termed GPΔmucΔw (Fig 2A). Indeed, binding to GPΔmucΔw is lost for only the four anti-wing mAbs, whilst the remaining six mAbs against different epitopes do bind GPΔmucΔw (S2 Fig). No definitive epitope information could be identified by pepscan for the remaining 6 antibodies, suggesting that these mAbs bind conformational epitopes.

### Sequence analysis of the GP2 wing

Sequence alignment of MARV GP residues 449–471 reveals that while Ci67, Musoke and Angola are completely conserved in this region, Ravn has 4 unique residues. The most notable change is residue 465, which is a Glu (E) in Ravn but a Lys (K) in the other strains (Fig 2B). Wing mAbs 54G2 and 30G3 are specific for MARV Ravn. Correspondingly, ELISA data comparing binding of wild-type Ravn GPΔmuc to E465K Ravn GPΔmuc confirm that the presence of Lys at position 465 (as exists in other strains of MARV) likely hinders binding of 54G2 and 30G3. 30G5, however, still retains some binding to E465K, while 30G4 is unaffected by this mutation, retaining binding at 2 and 0.2ug/ml (Fig 2C). These results agree with pepscan results (based on 15-mer peptides overlapping by 5 amino acids) which define the epitope for 30G4 as slightly shifted away from position 465, towards the N-terminus of GP2 (Fig 2B). This shift may explain why 30G4 is the most cross-reactive of the 4 anti-wing mabs (Fig 1C).

### *In vitro* neutralization

**Fig 3:**
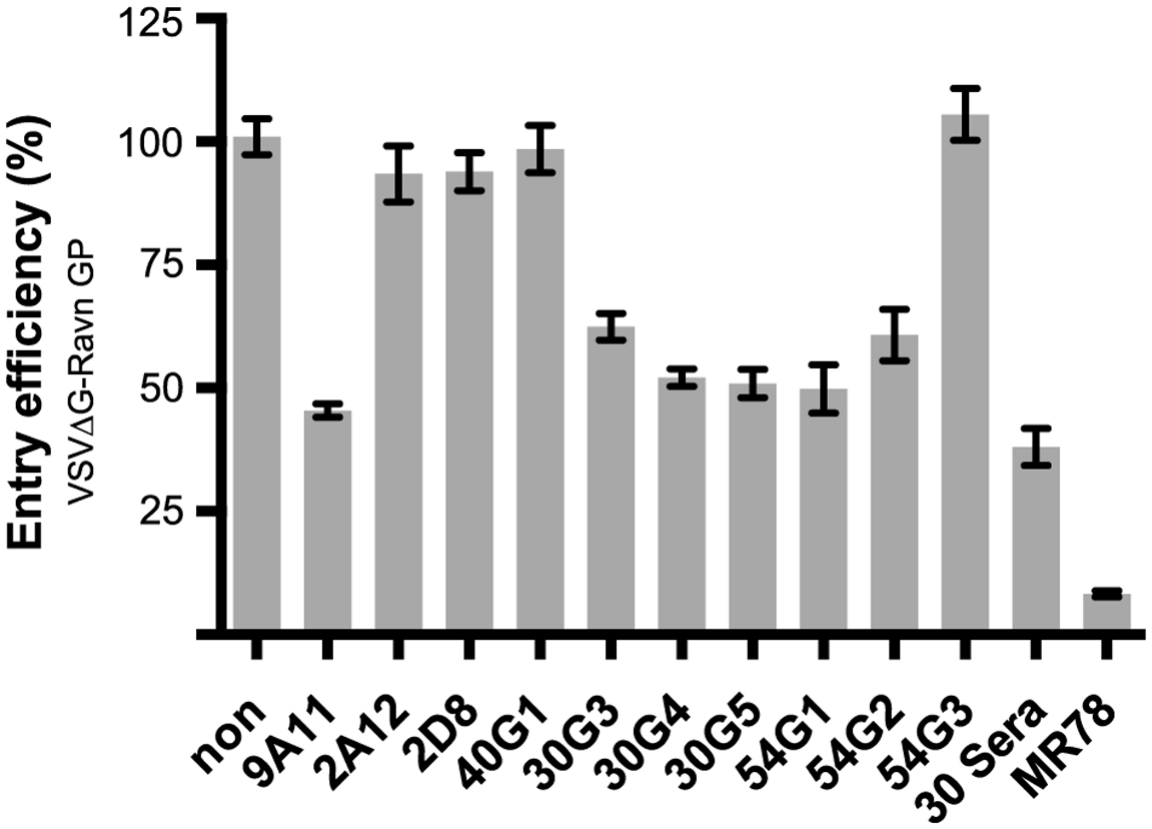
*In vitro* neutralization activity. Neutralization potency of mAbs against VSV pseudotyped with MARV Ravn GP (VSVΔG Ravn GP) in Vero cells. VSVΔG MARV Ravn GP was incubated with 50μg/ml of the indicated mAb for 1 hour before infection, and entry efficiency was calculated based on GFP expression. Positive control is human survivor mAb MR78.

Antibodies were screened for *in vitro* neutralization using a VSV-pseudovirus containing MARV Ravn GP on the surface. Six of the ten mAbs exhibit partial neutralization at the highest concentration tested (50ug/ml), reducing entry by 35–55%. The remaining four mAbs do not neutralize (Fig 3). Notably, all five GP2-directed mAbs produced in this study exhibit some neutralization, while only one GP1-directed mAb, 9A11, inhibits entry of Ravn GP pseudovirions. Polyclonal sera from mice that yielded the 30 series mAbs (30G3, 30G4 and 30G5) reduces entry by only about 60%, suggesting that mAbs 30G3, 30G4, and 30G5 represent the maximum potency of the polyclonal population (Fig 3). Human survivor mAb MR78 was used as a positive control and reduces pseudovirion entry by almost 95%.

### *In vivo* protection

**Fig 4:**
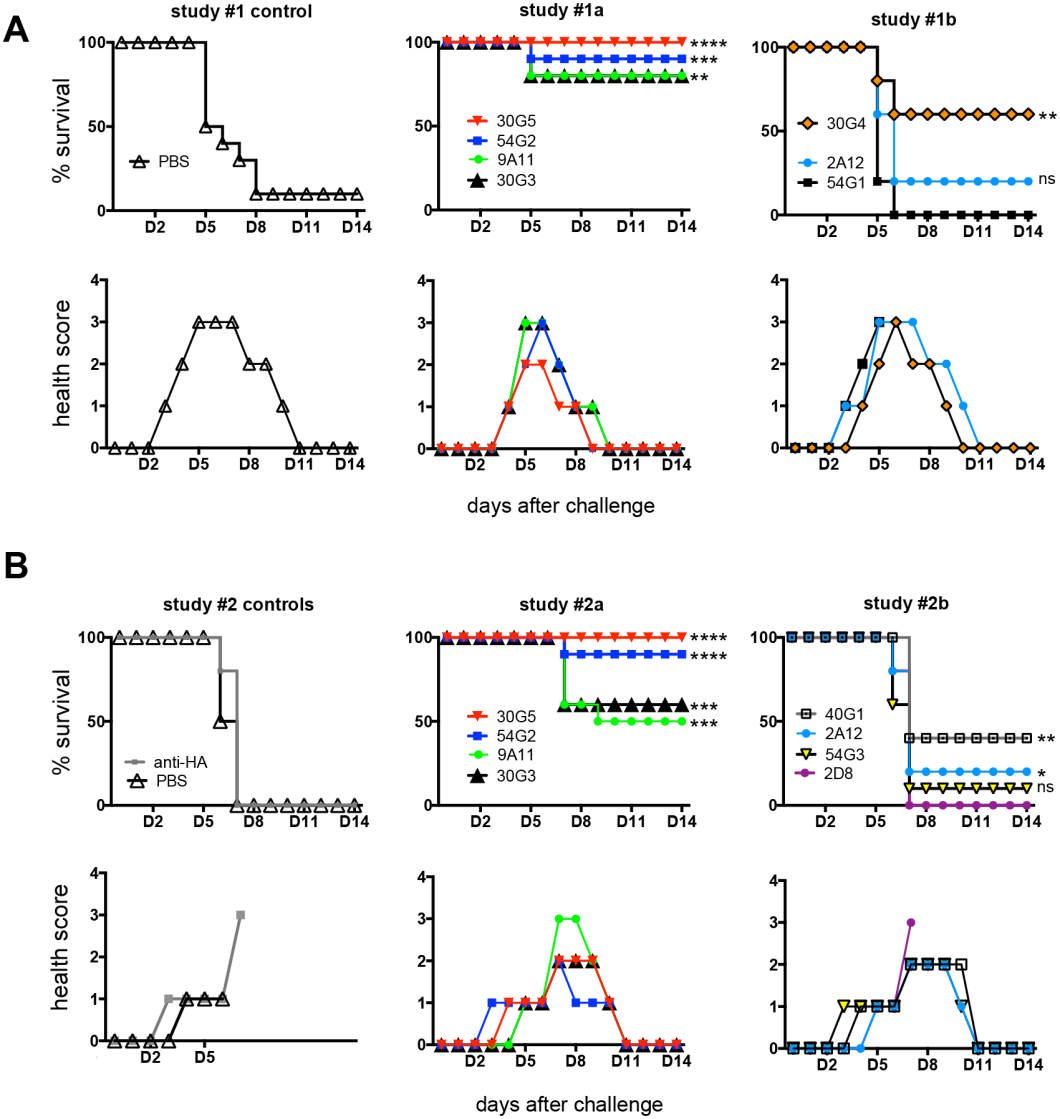
*In vivo* survival data. Groups of BALB/c mice at 10 animals per group were injected with individual mAbs one hour after challenge with mouse-adapted MARV Ravn virus. Two separate studies are represented; treatment groups are broken up into 3 or 4 mAbs to simplify survival and health score graphs. Studies continued for 28 days total, however no additional changes were observed beyond day 14. Asterisks represent P value summaries with non-significant curves labeled ns. (A) Study #1. PBS control survival is 10%. (B) Study #2. PBS control survival is 0%.

All mAbs were evaluated for *in vivo* protection using BALB/c mice challenged with a lethal dose of MARV virus [45]. One hour after challenge with 1,000 pfu mouse-adapted MARV Ravn, mice were treated IP with 500 μg purified mAb. Two separate studies were performed, with half of the mAbs repeated in both studies. Control animals in study #1, treated with PBS, exhibited 1/10 survival (Fig 4A). Both control groups in study #2, treated with PBS or anti-HA mAb, exhibited 0/10 survival (Fig 4B). MARV mAb treatment groups varied widely in efficacy, ranging from 0–100% protection. All four mAbs against the GP2 wing were found to be moderately or highly protective: mAb 30G3 conferred 70% survival (14/20), mAb 30G4 60% survival (6/10), mAb 30G5 100% survival (20/20), and mAb 54G2 90% survival (18/20). Monoclonal antibody 9A11, against GP1, conferred 65% survival (13/20) (Fig 4A and 4B). Other mAbs against the GP1 core exhibited 0–40% survival; of these, only 40G1 offered strongly significant protection (P value 0.0029). The only mAb against a GP2 epitope other than the wing, mAb 54G1, exhibited zero protection (0/10) (Fig 4A). In both studies, mice in all treatment groups displayed an elevation of disease score by Day 4 (Fig 4A and 4B), and there were no significant differences in weight loss between treatment groups and control groups. In study #1, 30G5-treated mice faired only modestly better than the other groups, reaching a disease score maximum of 2 and fully recovering by Day 9 (Fig 4A).

### Cross-reactivity with ebolavirus GPs

**Fig 5:**
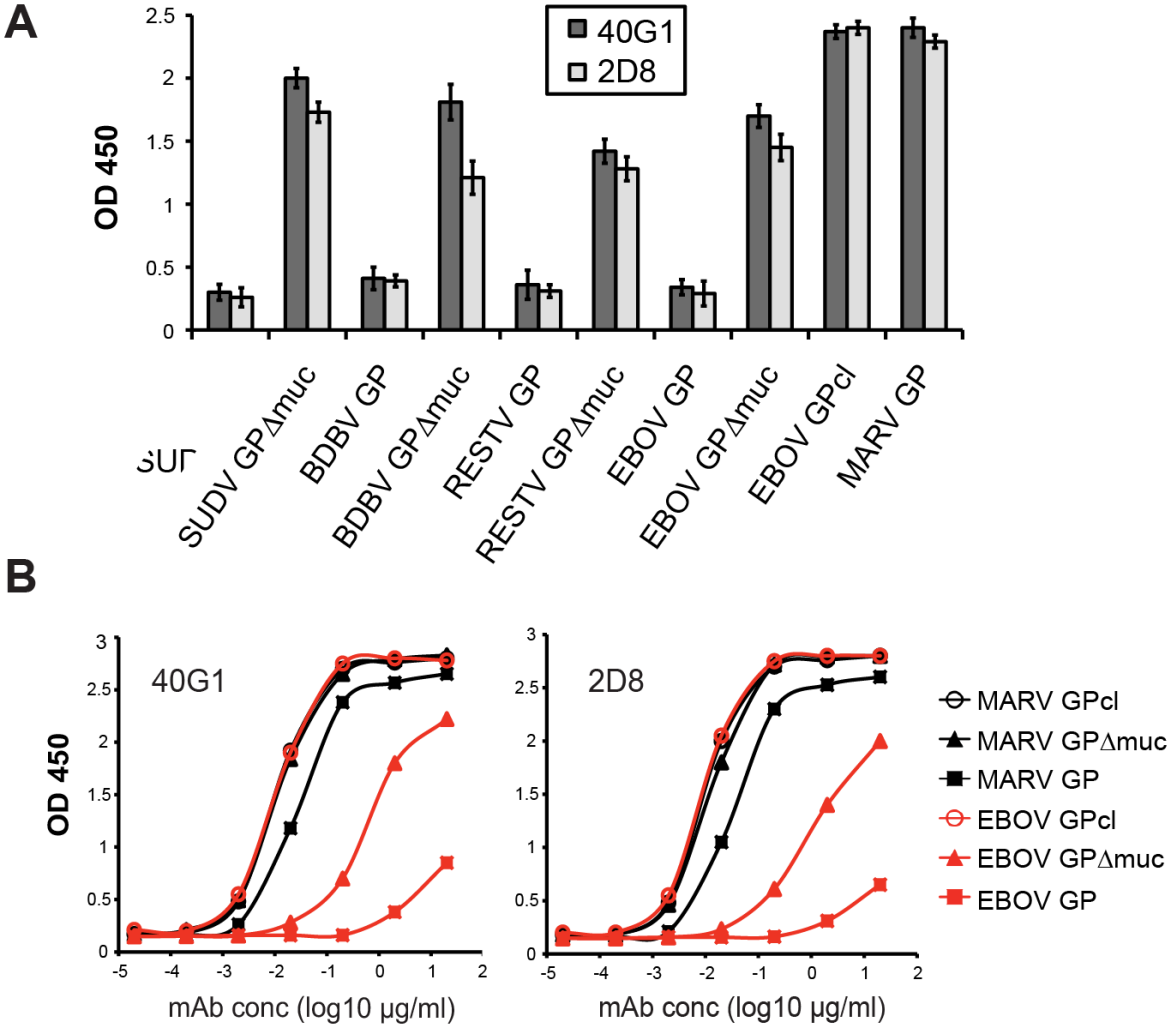
Filovirus GP cross-reactivity of 40G1 and 2D8. (A) Reactivity of 40G1 and 2D8 mAbs to GP antigens determined by ELISA at 5μg/ml. SUDV, Sudan virus; BDBV, Bundibugyo virus; RESTV, Reston virus. (B) Binding curves determined by ELISA with mAb serial dilutions starting at 20μg/ml. Note that MARV GPΔmuc, MARV GPcl, and EBOV GPcl curves overlay in both graphs.

Two of the highly cross-reactive MARV antibodies, mAbs 40G1 and 2D8, also exhibit binding to Ebola, Sudan, Bundibugyo and Reston virus mucin-deleted GPs by ELISA (Fig 5A). Binding curves show that the affinity of 40G1 and 2D8 for mucin-containing EBOV GP is weak, affinity for GPΔmuc is stronger, and binding to EBOV GPcl (the receptor-binding competent core) is strongest and equal to that of MARV GPcl (Fig 5B). Hence, the 40G1 and 2D8 epitopes are conserved across the filovirus family, exposed on all versions of Marburg virus GP, but masked on ebolavirus GP by the mucin-like domain and the glycan cap.

### Structural studies

**Fig 6:**
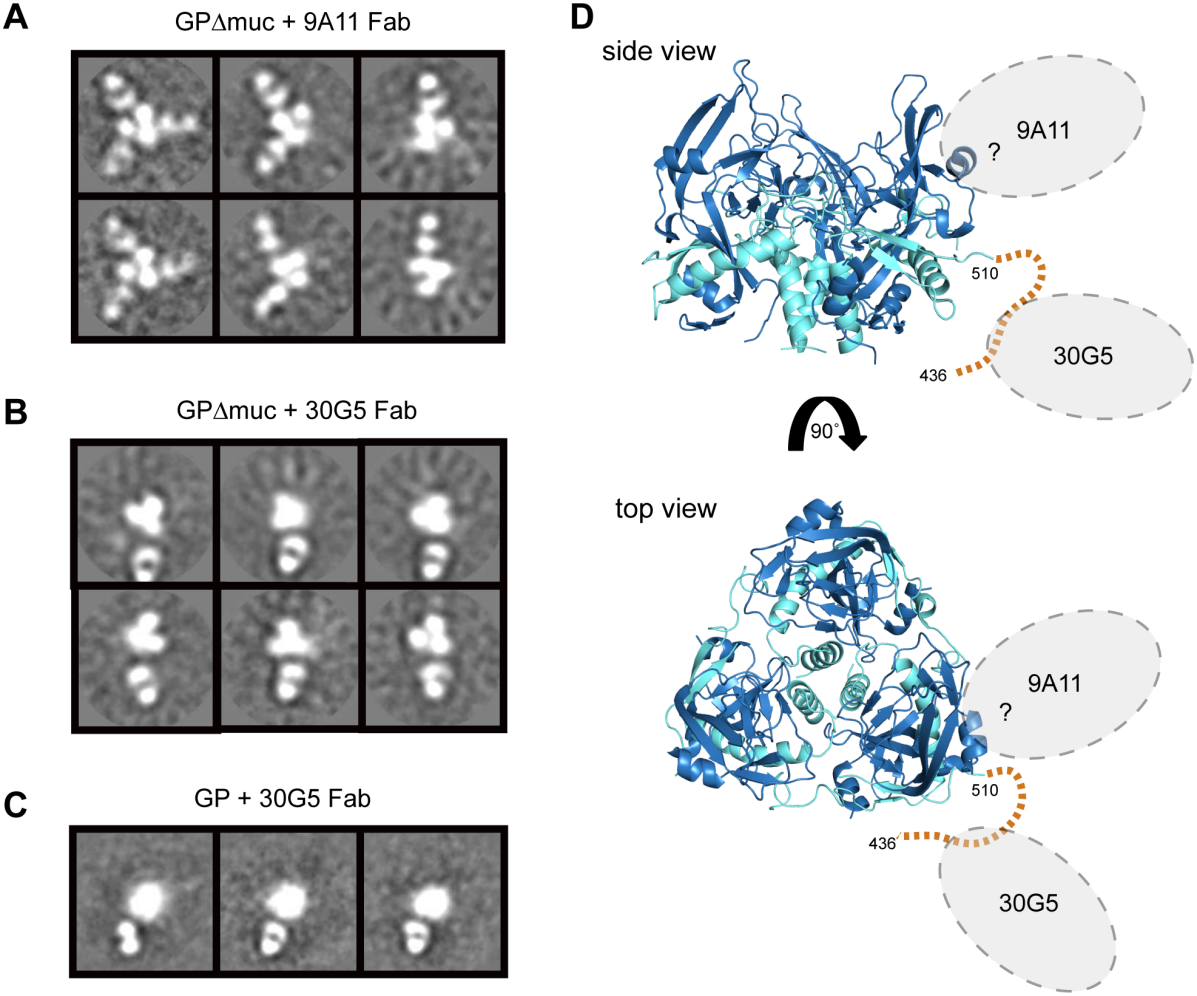
Negative stain EM and modeling of Fabs bound to MARV GP. Representative 2D class averages of MARV Ravn GP:Fab complexes (A) GPΔmuc + 9A11 (B) GPΔmuc + 30G5 (C) GP + 30G5. (D) The crystal structure of MARV GPcl is shown with the unresolved GP2-wing region (436–510) depicted by a dashed orange line. The footprints of 9A11 and 30G5 Fabs are unknown; possible binding areas are highlighted by gray dotted ovals. For simplification, Fab binding regions are highlighted on only one monomer of the trimer in each view.

Single particle electron microscopy of the most protective anti-GP1 (9A11) and anti-GP2 (30G5) antibodies was performed in complex with purified antigen. Negative stain 2D class averages of 9A11 Fabs in complex with MARV Ravn GPΔmuc show one, two, or three Fabs bound to the dense trimeric GP core (Fig 6A). In contrast, 2D class averages of the anti-GP2 wing mAb 30G5 in complex with MARV Ravn GPΔmuc show a single Fab bound to GP, at a distance further away from the high density GP trimer (Fig 6B). Deuterium exchange mass spectrometry (DXMS) studies suggest this GP wing region is unstructured and likely flexible (S2 Fig). To ensure that the wing epitope is not artificially positioned in GPΔmuc as compared to the biologically relevant mucin-containing GP, we also performed EM with 30G5 Fab in complex with the complete ectodomain of MARV Ravn GP. Images obtained were similar to those with GPΔmuc, with only one Fab binding per trimer (Fig 6C). Likely footprints of Fabs 9A11 and 30G5 are drawn onto the MARV Ravn GPcl crystal structure (Fig 6D).

## Discussion

In this study, a small panel of mAbs targeting MARV GP were isolated from immunized mice. Those that conferred the greatest *in vivo* protection are directed against a novel “wing” domain on MARV GP2. This wing region is a MARV-specific portion of the mucin-like domain attached to GP2. Such an epitope does not exist in ebolaviruses because the entire mucin-like domain is attached to GP1. Although this study size was small, we note that GP2 wing-directed mAbs were only obtained when mice were immunized with mucin-deleted Ravn GP. It may be tempting to assume that this epitope is masked by the mucin-like domain; however, anti-wing mAbs are able to access their epitope on mucin-containing GP, neutralize pseuodviruses bearing mucin-containing GP and provide *in vivo* efficacy when challenged with Marburg virus. We believe that the elicitation of anti-wing antibodies when using Ravn GPΔmuc may instead result from the greater homogeneity and stability of Ravn GPΔmuc over other MARV antigens. A seven-year protein engineering effort in our laboratory to identify crystallizable versions of MARV GP indeed found that GPs produced from strain Ravn are the most homogenous, and have a lesser tendency to aggregate than those from other strains of MARV [16]. The homogeneity may have lead to improved presentation of this protective epitope within this study.

It is interesting to note that among this panel of murine mAbs and the recently published panel of human survivor mAbs [30], no antibodies that bind the GP1- and GP2-containing base of MARV GP were identified. The “base” of GP is a common site of neutralization for the ebolaviruses and is the epitope target of anti-EBOV neutralizing antibodies KZ52 [15], 2G4 and 4G7 [46], as well as the anti-SUDV mAb 16F6 [47]. Perhaps the presence of the flexible GP2-wing in MARV blocks access to this site on the GP core. Nonetheless, antibodies directed against the GP wing itself do have the potential to be fully protective, and represent a novel epitope in MARV for therapeutic cocktail design. The most protective of these mAbs, 30G5, is promising but only binds with high affinity to the GP from Ravn, and hence, protection by 30G5 against other MARV strains may be limited. In contrast, monoclonal antibody 30G4 only confers 60% efficacy, yet cross-reacts with mucin-containing GPs from four strains of MARV. However, 30G4 is a murine IgG1, an isotype that typically exhibits weaker immune effector activity than murine IgG2a [48]. Replacement of the constant domain framework may improve its *in vivo* efficacy.

In this panel, two mAbs against GP1 were identified which also bind the GP cores of ebolaviruses. These antibodies, 40G1 and 2D8, bind all MARV GPs, but only bind Ebola, Sudan, Bundibugyo and Reston GP from which the mucin-like domain is deleted (Fig 5). Hence, these highly conserved epitopes are exposed on marburgvirus GPs, but masked on ebolavirus GPs. These observations parallel those obtained from a panel of anti-MARV GP antibodies isolated from a human survivor [30], and support structural observations that the orientation of the mucin-like domains differs between EBOV and MARV [16]. Indeed, no cross-filovirus anti-GP antibody (reactive to both ebola and marburg) has yet been elicited by an ebolavirus GP immunogen, nor has any such antibody yet been isolated from an ebolavirus survivor. Although the filovirus cross-reactive mAb 40G1 confers only 40% survival, 40G1 or another antibody like it [30] may be useful in an immunotherapeutic cocktail because a highly conserved epitope would likely be less subject to antigenic escape.

Antibody 9A11 is also directed against GP1 but its pattern of binding is distinct from 40G1 and 2D8. 9A11 is the only mAb in this panel that has a lower affinity to GPcl than GP or GPΔmuc. This suggests that the epitope of 9A11 is partially lost upon cleavage and that 9A11 could be similar to a glycan cap binder like 13C6 or 1H3 for EBOV GP [46]. Unfortunately, due to the single preferred orientation of GPΔmuc + 9A11 Fab particles on negative stain EM grids, a high-resolution reconstruction could not be determined, and better understanding of the 9A11 epitope awaits further study. 9A11 affords 65% protection *in vivo* and is highly cross-reactive among MARV ectodomain GPs.

For Ebola virus, *in vitro* neutralization is not necessarily an effective predictor of *in vivo* protection. One anti-EBOV cocktail is composed entirely of non-neutralizing or weakly neutralizing mAbs, yet still confers *in vivo* protection, presumably by recruiting immune effector function [44,49]. More recent cocktail formulations have included a mix of neutralizing and non-neutralizing antibodies [50]. In this study of mAbs against MARV, none of the mAbs offered significant *in vitro* neutralization, yet several did confer partial to complete *in vivo* protection against MARV one hour after challenge. Although this study is limited in scope, we note that among this set of antibodies, those that exhibited *in vitro* neutralization also conferred the best *in vivo* protection. (There was only one mAb that weakly neutralized but offered no protection, mAb 54G1). Future studies, performed at longer time periods after challenge and with lower treatment doses, will test the limits of efficacy of the individual mAbs. Promising mAbs could then be evaluated in non-human primates (NHPs) to predict therapeutic potential in humans.

In this work, we provide biochemical and structural mapping of antibody epitopes on MARV GP, and analyze the conservation of these epitopes among different strains of MARV. We find antibodies against a novel GP2 “wing” epitope that confer 90–100% protection *in vivo*, and two mAbs against different sites in GP1 that confer 40% and 65% protection. mAb cocktails are thought to be most effective when the component antibodies display synergistic effects. Combining mAbs with non-overlapping epitopes can significantly increase the overall potency of the cocktail over the individual mAbs alone [37,39], and can mitigate antigenic escape by the virus [51]. The panel of antibodies described here, although limited in number, provides three possible components of an anti-MARV immunotherapeutic cocktail: an anti-GP1 core mAb such as 40G1 (or a neutralizing MR mAb), the anti-GP1 mAb 9A11, and an anti-GP2 wing mAb such as 30G4 or 30G5. Future studies will determine the limits of protection and therapeutic potential of these antibodies when delivered in combination.

## Materials and Methods

### Ethics statement

This study was approved and carried out in accordance with protocols provided by the Institutional Animal Care and Use Committee (IACUC) at TSRI, Emergent Biosolutions, NIAID, and USAMRIID. Research at USAMRIID was conducted in compliance with the Animal Welfare Act and other federal statutes and regulations relating to animals, and adhered to principles stated in the Guide for the Care and Use of Laboratory Animals, National Research Council, 1996.

### Immunogen preparation

#### Marburg Angola, Ravn and Ci67 GPΔmuc (TSRI)

Marburg virus GP immunogens used to raise antibodies at Emergent were designed and produced at TSRI. DNA encoding the MARV GPΔmuc ectodomain (residues 1–636 with a mucin deletion of residues 257–425) was cloned into a derivative of the Invitrogen pDisplay vector. In this derivative vector, the PGDFR sequence is replaced by a C-terminal purification tag (either an HA or strep tag). Large-scale production was performed by PEI transfection (Polysciences, Inc MW 25,000) of plasmid into 70% confluent HEK293 GnTI-/- cells (ATCC) in Corning 10-layer Cellstacks. Supernatants were harvested four days post-transfection, concentrated with a Centramate tangential flow system, and affinity purified using Streptactin (Qiagen) or anti-HA 3F10 (Roche) affinity resin. Trimeric GPΔmuc was then isolated by S200 size exclusion chromatography (SEC) in 10 mM Tris, 150 mM NaCl, pH 7.5 (1x TBS). In order to improve furin cleavage processing during expression, which decreased aggregation and improved yield of purified trimers, bulky hydrophobic residues near the furin cleavage site were mutated in the GPΔmuc constructs. Ravn GPΔmuc mutations included F438L, W439A, F445G, F447N and Angola GPΔmuc mutations included W439V, M444A, F445G.

#### Marburg Musoke and Angola GPΔmuc (IBT)

Marburg virus GPΔmuc antigens used to raise antibodies at IBT were produced at IBT. Musoke GPΔmuc (1–636 Δ264–425) was produced by PEI transfection of HEK293T cells with the derivative pDisplay plasmid containing a C-terminal HA-tag. Protein was purified by anti-HA 3F10 affinity (Roche) followed by lectin affinity and Superose 6 size exclusion chromatography (GE Healthcare). Angola GPΔmuc (1–636 Δ264–425) was produced by baculovirus infection (Bac-to-Bac, Invitrogen) of Sf9 cells (Invitrogen) according to the manufacturer’s protocol, and purified via a C-terminal Hisx6 tag on Ni2+-NTA Sepharose resin (GE healthcare).

### Monoclonal antibody (mAb) production

#### Production of 30G3, 30G4, 30G5, 40G1, 54G1, 54G2, and 54G3

Six week-old BALB/c mice were injected subcutaneously (SC) with 20 μg (in 100 μl volume PBS) of purified MARV GPs in Freund’s Complete Adjuvant (CFA; Brenntag Biosector). Additional boosts were injected intraperitoneally (IP) on day 32 and 56 with 20 μg of the same GP in Incomplete Freund’s Adjuvant (IFA; Brenntag Biosector). Thereafter mice received a final push of 10 μg purified GP (in PBS by IP) before conducting fusions. Standard protocols were used to produce hybridoma cell lines [52], and monoclonal antibodies specific to GPΔmuc antigen were purified on Protein G resin. Mice immunized with Ravn GPΔmuc raised antibodies 30G3, 30G4 and 30G5. Mice immunized with Ravn GPΔmuc, then boosted two times with a complex of Ravn GPΔmuc bound to 30G4 Fab, raised antibodies 54G1, 54G2 and 54G3. Mice immunized with Angola GPΔmuc yielded antibody 40G1. Immunization of mice at Emergent was performed according to Animal Use Protocols (AUP) approved by the Protocol Management and Review Committee (PMRC), University of Manitoba.

#### Production of 2A12 and 2D8

Six week-old BALB/c mice were immunized intramuscularly (IM) three times with 50 μg purified MARV GPs in Glucopyranosyl Lipid Adjuvant (GLA) adjuvant at 2 week intervals, and boosted intravenously (IV) with 50 μg antigen 3 days before harvest of spleen/lymph nodes for fusions. Standard protocols were used to produce hybridoma cell lines [52], and monoclonal antibodies specific to GPΔmuc antigen were purified on Protein G resin. Mice immunized with Musoke GPΔmuc yielded antibody 2A12. Mice immunized with Angola GPΔmuc yielded antibody 2D8. Immunization of mice at IBT was performed according to AUP approved by Noble Life Sciences IACUC.

#### Production of 9A11

Immunization of mice was performed by Bio-Quant Inc (San Diego, CA). Six week-old BALB/c mice were injected subcutaneously (SC) with 20 μg of purified Ci67 GPΔmuc in CFA followed by additional boosts at 3 week intervals with 20 μg antigen (10 μg in IFA by IP and 10 μg in IFA by SC) before conducting fusions. Standard protocols were used to produce hybridoma cell lines, and mAb 9A11 was purified on Protein G resin.

### Antibody characterization

#### Purified filovirus GP antigen preparation

All filovirus ectodomains for ELISA, western blot, EM, or DXMS were produced at TSRI in *Drosophila* S2 cells [16], with the exception of Musoke GP, which was produced by IBT in Sf9 cells (as described above for Musoke GPΔmuc). Briefly, Effectene Reagent (Qiagen) was used to transfect S2 cells with pMTpuro plasmids containing a strep-tagged filovirus GP gene of interest, followed by stable selection of transfected cells with 6 μg/ml puromycin in Insect XPRESS protein free medium (Lonza). Secreted GP ectodomain expression was induced with 0.5mM CuSO4 and supernatants harvested after 4 days. Proteins were affinity purified using Streptactin resin (Qiagen), followed by purification via Superdex 200 SEC in 1x TBS. The cleaved “core” ectodomain for MARV (MARV GPcl) was produced by incubating 1mg Ravn GPeΔmuc with 0.01 mg trypsin (Sigma) at 37°C for 1 hour in TBS pH 7.5, followed by S200 SEC purification. The cleaved “core” ectodomain of EBOV (EBOV GPcl) was produced by incubating 1mg EBOV GPeΔmuc with 0.02mg thermolysin (Sigma) overnight at room temperature (RT) in TBS buffer plus 1mM CaCl_2,_ followed by S200 SEC purification. SDS-PAGE gels comparing purity and molecular weight of several antigens in shown in S1 panel B.

#### VLP preparation

Virus-like particles were produced by co-transfection of HEK293T cells with pCAGGS plasmids expressing full-length MARV Ravn GP or MARV VP40. Supernatants were harvested after sixty hours, VLPs pelleted down at 13,000 xg for 90 minutes, washed with PBS, and re-pelleted. VLP pellets were then gently resuspended in PBS containing 0.1% NP40 and 0.1% Triton X100, diluted 1:10 with PBS, and used as coating antigen for ELISA.

#### Western blotting

Purified MARV Ravn GP reduced and non-reduced samples were run on 10–15% SDS-PAGE gels and transferred onto PVDF Immobilon membranes (Millipore). Membranes were blocked overnight in 5% milk (BioRad Blotting grade) PBS-0.05% Tween 20 (PBS-T), incubated for 1hour at room RT with anti-MARV mabs at a concentration of 2ug/ml in 1% milk PBS-T, washed with PBS-T, then incubated with goat anti-mouse (or anti-human for MR78) alkaline phosphatase (AP) conjugated antibody at a 1:2000 dilution. AP activity was detected with SigmaFast BCIP/NBT substrate.

#### Recognition of various forms of GP and cross-reactivity by ELISA

To determine half maximal effective concentrations, or EC50s, mAbs were tested for binding to GP (1–636), GPΔmuc (1–636 Δ257–463), and GPcl of MARV Ravn at a concentration range of 10μg/ml to 0.01ng/ml using 10-fold serial dilutions. Data was analyzed using Graphpad Prism 6.0 software. To determine cross-reactivity, mAbs were tested for binding to MARV Angola, Musoke, Ci67, and EBOV GP (1–636) at 10, 1, 0.1 and 0.01μg/ml. Because mucin-containing Musoke and Ci67 GPs readily aggregate, binding in Fig 1C is reported as high, medium, or low rather than as a quantitative EC50 value. Antibodies 2D8 and 40G1 were further analyzed for binding to the GPΔmuc antigens of EBOV (1–637 Δ312–462), SUDV (1–637 Δ314–472), BDBV (1–637 Δ314–463) and RESTV (1–637 Δ316–470), as well as to EBOV GPcl. Binding curves for 2D8 and 40G1 were determined at a concentration range of 20μg/ml to 0.017ng/ml using 3-fold serial dilutions. ELISAs were performed as follows: Corning 96-well high-binding microtiter plates were coated with filovirus GP antigens, blocked with 3% BSA in PBS 1 hour at RT, and incubated with anti-MARV mAbs in 0.3% BSA 1 hour at RT. Plates were then incubated with 1:2000 goat anti-mouse IgG (H+L) HRP conjugated secondary (Thermo Scientific) in 0.3% BSA 1 hour at RT. (Plates were washed between each step with PBS containing 0.05% Tween 20). Color development was produced with TMB substrate (Thermo Scientific), stopped with 1N sulfuric acid, and quantified by measuring absorbance at 450nm.

#### Pepscan and GP2 wing analysis

In an attempt to map linear epitopes, all mAbs were tested by ELISA pepscan against synthetic 15-mer peptides designed from Ravn GP sequences, overlapping by 5 amino acids. Pepscan was repeated for mAbs 9A11, 2A12, 2D8 and 40G1 against peptides designed from Musoke GP sequences. As a control for GP2 wing pepscan defined epitopes, additional ELISAs were performed with a MARV GP lacking both the GP1 MLD (Δ257–425) and the GP2 wing (Δ436–483), termed GPΔmucΔw. GP2 wing-directed antibodies were further evaluated for binding to both wild-type Ravn GPΔmuc and Ravn GPΔmuc containing a point mutant (E465K). Coating antigens for the point mutant ELISAs were produced in HEK293T cells to represent a mammalian glyco-profile in and around the GP2 wing.

#### Pseudovirus neutralization assays

Vesicular stomatitis virus (VSV) pseudovirions containing a GFP gene in place of the VSV envelope glycoprotein gene (VSVΔG) and bearing the full-length glycoprotein of MARV Ravn were generated as previously described [53]. Pseudovirions were incubated with anti-VSV G mAb (a gift from A. Takada) for 1 hour at RT, then incubated with 50 μg/ml of each anti-MARV GP mAb in DMEM-10% FBS (Gibco) for an additional hour at RT. Pseudovirion/mAb complexes were added to Vero cell (ATCC) monolayers in 96-well plates at a multiplicity of infection (MOI) between 0.01 and 0.03. After 48 hours, infection was evaluated by counting GFP-expressing cells. Experiments were performed in triplicate and standard deviations displayed. All mammalian cell lines used in this study tested negative for mycoplasma contamination at TSRI.

### *In vivo* testing

#### Animal work

All procedures with infectious Marburg viruses were performed in a biosafety level 4 (BSL4) facility at USAMRIID. Male and female BALB/c mice between 6 and 10 weeks of age were challenged intraperitoneally (IP) with 1000 plaque-forming units of mouse-adapted MARV Ravn [45] in two separate studies. One hour post-exposure, the mice were treated IP with 500 μg of purified monoclonal anti-MARV GP antibody in PBS (1.0 mg/ml) or PBS alone. Study two included an additional negative control group treated with 500 μg of anti-HA IgG in PBS (1.0mg/ml). Each test group consisted of 10 animals for a total of 210 mice. All antibodies were blinded by IBT before submission to USAMRIID researchers. Animals were weighed and monitored daily over a 28 day period post-challenge, at which point mice were euthanized in accordance with an IACUC-approved protocol. Once animals were symptomatic, they were examined twice per day. Health was scored using the following parameters: 0 = normal, 1 = reduced grooming/ruffled fur, 2 = subdued, 3 = lethargic/hunched posture (provide DietGel for hydration), 4 = unresponsive; euthanize. Health scores for the 54G3 and 2A12 treatment groups in Fig 4 are shown for the one or two animals that survived, respectively. No animals were excluded from analysis and the experiments were not randomized. All BALB/c mice used in these experiments were obtained from the Frederick Cancer Research and Development Center, National Cancer Institute (Frederick, MD).

#### Statistical analysis

Graphpad Prism 6.0 software was used to calculate P values using the Log-rank Mantel Cox test. Each treatment group was compared to the corresponding PBS control for either Study #1 or #2. P values > 0.05 are considered non-significant (ns).

### DXMS

Purified Ravn GPcl was evaluated by Deuterium Exchange Mass Spectrometry (DXMS) as previously described [24].

### Electron microscopy and image processing

For negative stain EM analysis, MARV Ravn ectodomains were produced in *Drosophila* S2 cells as described above. Fab 30G5 and 9A11 fragments were generated by standard papain digestion (Sigma) of IgG and purified by Mono Q (GE Healthcare) ion-exchange chromatography. Five molar excess Fab was added to trimeric GPΔmuc or GP and allowed to bind overnight at 4°C. Complexes were diluted to 0.03mg/ml in TBS buffer and deposited onto to carbon-coated 400 copper mesh grids which had been plasma cleaned for 20 sec (Gatan) and stained for 30 sec with 4 μL of 2% uranyl formate. The stain was blotted off the edge and the grid was allowed to dry. Data were automatically collected with Leginon [54] using a FEI Tecnai F20 electron microscope operating at 120 keV with an electron dose of 30 e^-^/Å^2^ and a magnification of 52,000X that resulted in a pixel size of 2.65 Å at the specimen plane when collected with a Spirit 4k x 4k CCD camera (for 30G5) and 2.05 Å at the specimen plane when collected with a Tietz 4k x 4k CCD camera (for 9A11). Images were acquired at a constant defocus value of -1.3 μm at various tilt angles from 0 to 50°. Particles were picked automatically using DoG Picker [55] and placed into a particle stack using the Appion software [56]. Reference-free 2D class averages were calculated by using particles binned by 2 with the Xmipp Clustering 2D Alignment software [57] and sorted into ~50–100 particles per class.

## Supporting Information

S1 Fig (A) Western blot of anti-GP MARV mabs incubated at 2μg/ml, against reduced (+ DTT) and non-reduced (- DTT) purified Ravn GP. (B) Non-reducing 10–15% SDS-PAGE gels of several purified MARV GP and Ebola antigens from S2 cells. Note Ravn GPcl runs larger than Ebola GPcl due to extra mass of the GP2 wing.
(TIF)

S2 Fig Reactivity of anti-GP MARV mabs by ELISA at 10μg/ml to purified GPΔmuc or GPΔmucΔw. Note only the four anti-wing mabs lose binding to GPΔmucΔw (refer to Fig 1A for construct schematic).
(TIF)

S3 Fig Purified Ravn GPcl was evaluated by Deuterium Exchange Mass Spectrometry (DXMS) and the GPΔmuc sequence used to analyze peptides. The GP2 wing region is outlined in an orange box. Peptide fragments in this region have very high levels of deuteration, indicating that the GP2 wing is solvent exposed, and likely unstructured.
(TIF)
