# Correction: Protective mAbs and Cross-Reactive mAbs Raised by Immunization with Engineered Marburg Virus GPs

**DOI:** 10.1371/journal.ppat.1005212

**Published:** 2015-10-09

**Authors:** Marnie L. Fusco, Takao Hashiguchi, Robyn Cassan, Julia E. Biggins, Charles D. Murin, Kelly L. Warfield, Sheng Li, Frederick W. Holtsberg, Sergey Shulenin, Hong Vu, Gene G. Olinger, Do H. Kim, Kevin J. Whaley, Larry Zeitlin, Andrew B. Ward, Cory Nykiforuk, M. Javad Aman, Jody D. Berry, Erica Ollmann Saphire

There is an error in the 18th author's name; the middle initial is missing. The correct name is Jody D. Berry. This author's affiliation is also incorrect.

The correct affiliation is: Cangene Corp, now a subsidiary of Emergent Biosolutions. Winnipeg, Manitoba, Canada.

This author's current address is: Proteos, Inc., Kalamazoo, MI
