# Supplementary figures and images for: Protective mAbs and Cross-Reactive mAbs Raised by Immunization with Engineered Marburg Virus GPs
Source: PLoS Pathog. 2015 Jun 26;11(6):e1005016. doi: 10.1371/journal.ppat.1005016 (PMC4482612; doi:10.1371/journal.ppat.1005016)

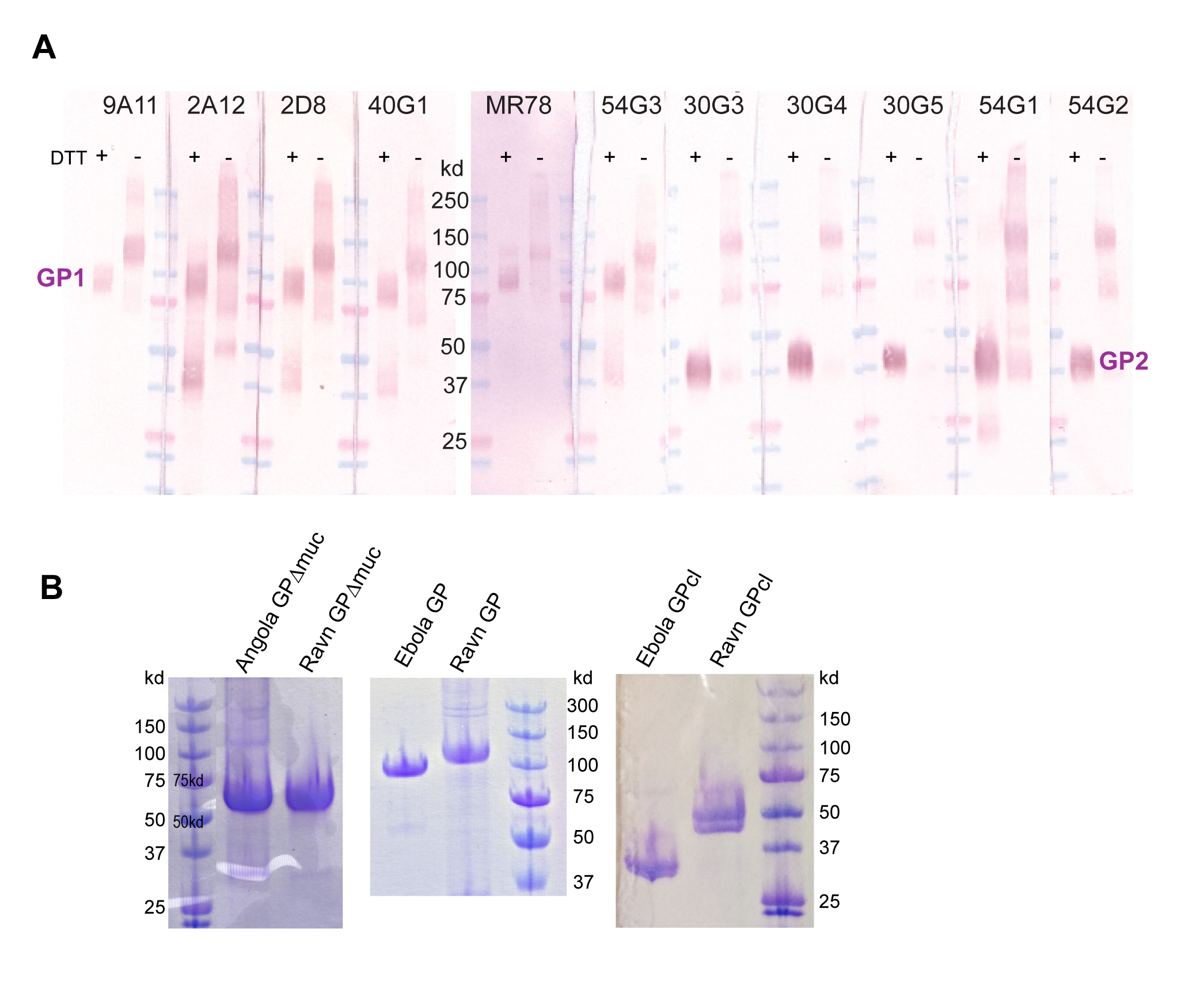

Supplement: S1 Fig — (B) Non-reducing 10–15% SDS-PAGE gels of several purified MARV GP and Ebola antigens from S2 cells. Note Ravn GPcl runs larger than Ebola GPcl due to extra mass of the GP2 wing. (TIF) [file ppat.1005016.s001.tif]

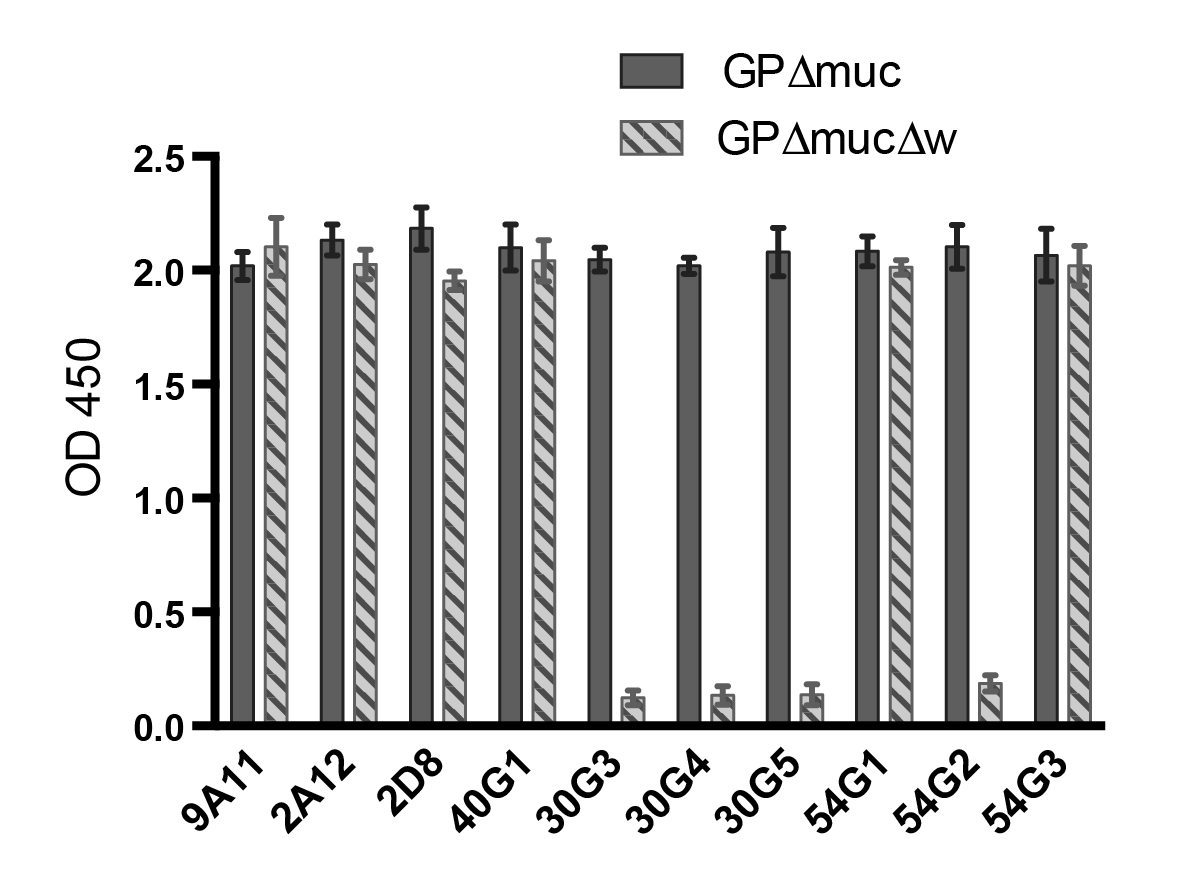

Supplement: S2 Fig — Note only the four anti-wing mabs lose binding to GPΔmucΔw (refer to Fig 1A for construct schematic). (TIF) [file ppat.1005016.s002.tif]

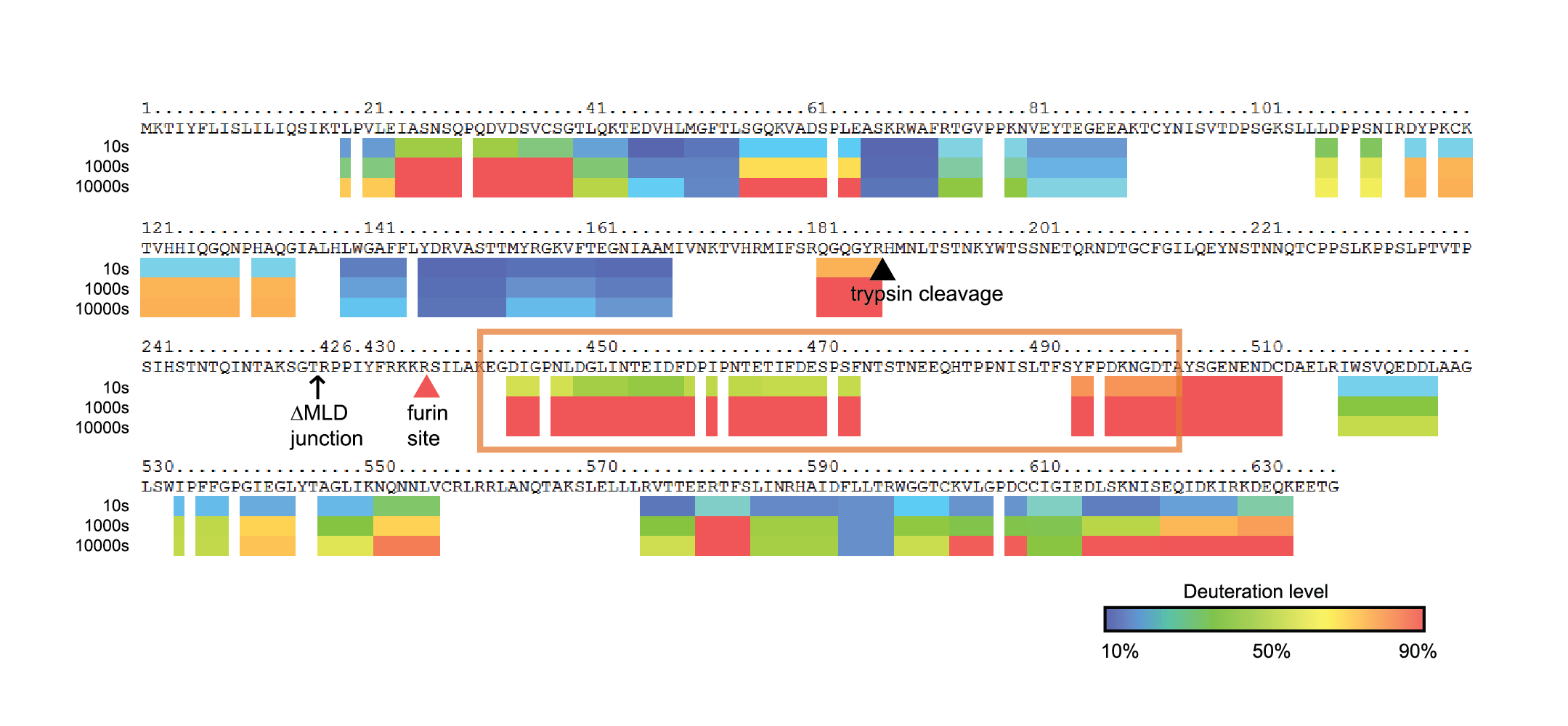

Supplement: S3 Fig — The GP2 wing region is outlined in an orange box. Peptide fragments in this region have very high levels of deuteration, indicating that the GP2 wing is solvent exposed, and likely unstructured. (TIF) [file ppat.1005016.s003.tif]
